# Sensing and Tactile Artificial Muscles from Reactive Materials

**DOI:** 10.3390/s100402638

**Published:** 2010-03-25

**Authors:** Laura Valero Conzuelo, Joaquín Arias-Pardilla, Juan V. Cauich-Rodríguez, Mascha Afra Smit, Toribio Fernández Otero

**Affiliations:** 1 Center for Electrochemistry and Intelligent Materials (CEMI), Universidad Politécnica de Cartagena, ETSII, E- 30203, Cartagena, Spain; E-Mails: laura.valero@upct.es (L.V.C.); joaquin.arias@upct.es (J.A.-P.); 2 Materials Department, Centro de Investigación Científica de Yucatán (CICY), C.P. 97200 Mérida, Mexico; E-Mails: jvcr@cicy.mx (J.V.C.-R.); mascha@cicy.mx (M.A.S.)

**Keywords:** conducting polymers, reactive materials, sensors, actuators, artificial muscles, tactile muscles, sensing actuators

## Abstract

Films of conducting polymers can be oxidized and reduced in a reversible way. Any intermediate oxidation state determines an electrochemical equilibrium. Chemical or physical variables acting on the film may modify the equilibrium potential, so that the film acts as a sensor of the variable. The working potential of polypyrrole/DBSA (Dodecylbenzenesulfonic acid) films, oxidized or reduced under constant currents, changes as a function of the working conditions: electrolyte concentration, temperature or mechanical stress. During oxidation, the reactive material is a sensor of the ambient, the consumed electrical energy being the sensing magnitude. Devices based on any of the electrochemical properties of conducting polymers must act simultaneously as sensors of the working conditions. Artificial muscles, as electrochemical actuators constituted by reactive materials, respond to the ambient conditions during actuation. In this way, they can be used as actuators, sensing the surrounding conditions during actuation. Actuating and sensing signals are simultaneously included by the same two connecting wires.

## 1. Introduction

Most of the technological advances developed by humans are inspired on natural systems, organs, functions or devices present in living creatures and developed through millions of years of biological evolution. Natural systems and devices combine efficiency with intellectual, scientific and technological elegance.

**Figure 1.**
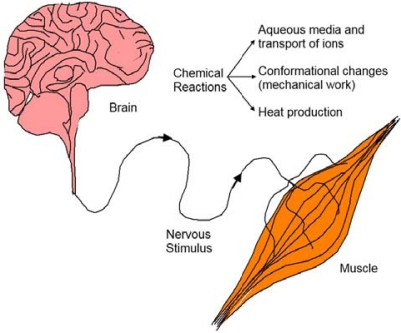
Actuation of natural muscles involves electric signals, chemical reactions, conformational movements, interchange of ions and water and heat production.

Accordingly, natural muscles are elegant devices developed to transform chemical energy into mechanical energy and heat [1]. With respect to actuation, the main difference between human technology and natural muscles is that during actuation the chemical composition changes inside the wet organ, meanwhile dry materials from artificial machines keep a constant composition. The actuation of a natural muscle (Figure 1) involves: (a) aqueous media, (b) electric pulses arriving from the brain (the pulse generator) to the muscle through the nervous system, (c) liberation of calcium ions inside the sarcomere, (d) chemical reactions, (e) conformational changes along natural polymeric chains (actin and myosin) with change of the sarcomere volume and (f) water interchange. This natural motor is able to perform elegant and gentle movements still not reproduced by any man-made motor. Moreover, the actuation of the muscle involves simultaneous sensing processes, providing living creatures with a perfect consciousness of characteristics of the mechanical movements and physical interactions between the organ moved by the muscle and its environment.

The chemical and physical processes linked to the electrochemistry of conducting polymers present a great deal of similarities with the above described biological processes, much more similar than those linked to any other artificial material [2–6].

## 2. Conducting Polymers: Classification

**Figure 2.**
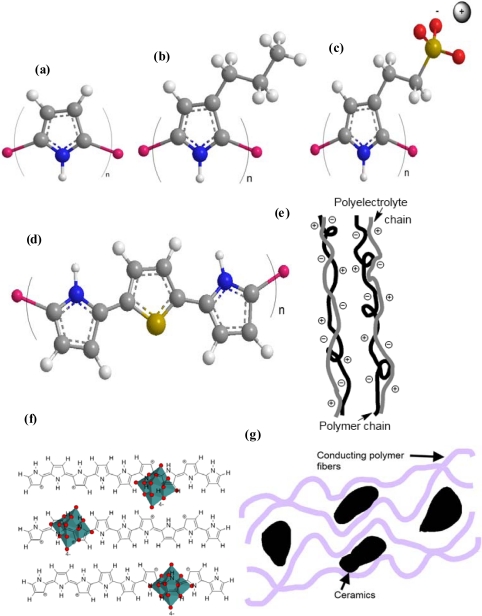
Classification of conducting polymers, (a) Basic conducting polymer, (b) substituted polymer, (c) self-doped polymer, (d) copolymer, (e) blend, (f) hybrid material and (g) composite.

Conducting polymers (CP) are classified into seven different families of materials (Figure 2) [2]:

- **Basic conducting polymers:** Polypyrrole [7–13], polyaniline [14–19], polythiophene [20–24], polyfurane [24–26], polyindole [24,27,28], *etc.*
- **Substituted polymers** [27,29–31]. One, or several, hydrogen atoms from the basic monomer was replaced by another chemical group. Hundreds of different chemical groups can be used to obtain new substituted monomers and polymers.
- **Self-doped polymers** [32,33]. One of the substituents is an ionic group.
- **Copolymers** [34–38]. Two or more monomers are present on the final polymer chain.
  **Polymer/macro-ion blends**. The synthesized oxidized material contains macro-ions, which are not interchanged during redox processes:
- **Polymeric or organic blends** [39–42]. The macro-ion is a polyanion or an organic macro-ion.
- **Hybrid materials** [43–45]. The macro-ion is inorganic
- **Composites** [46–50]: With different organic or inorganic materials.

Every component of a family originates, by electrochemical oxidation or reduction, hundreds of oxidized materials (polymer/counterion/solvent), depending on the salt used in the solution during the electrochemical reaction.

## 3. Electrochemical Behavior of Conducting Polymers in Solution

**Figure 3.**
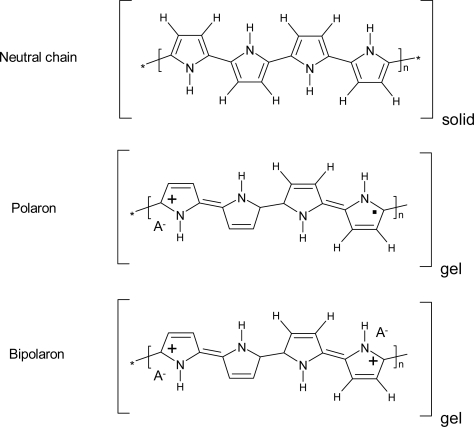
Transformation of the chemical bonds during oxidation to form polarons (radical cations). When the chain is saturated of polarons, the extraction of new electrons generates bipolarons (dications). Counterions (A^−^) penetrate from the solution for charge balance.

During electrochemical oxidation (or reduction) of neutral polymeric chains, positive (or negative) charges are stored along the polymeric chains (Figure 3) and balancing counterions are forced to penetrate from the solution [2–6,51,52] to generate a new material (polyelectrolyte).

Depending on the balancing ion, different materials (polyeletrolytes) are obtained. The emerging positive (or negative) charges on the chains induce strong polymer/solvent interactions forcing the entrance of solvent from the solution. A complex material, polymer/ion/solvent is obtained.

**Figure 4.**
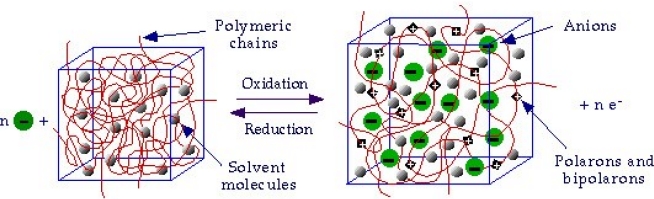
Schematic representation of the reversible volume change associated with the electrochemical reactions of polypyrrole in electrolytes (adopted from Reference [4] with kind permission from Springer Science and Business Media Media).

There exist two kinds of oxidation processes:

- **prevailing anion interchange**: The polymer swells during oxidation (Figure 4) and shrinks during reduction (accepted for families (a), (b), (f), and (g)) [14,53,54]:
  (1) $$\begin{array}{lll} {\left({pPy}^{0}\right)}_{s}+n{\left(A^{-}\right)}_{\mathrm{aq}}+m(\mathrm{Solvent}) & ↔ & {\left[{\left({pPy}^{n+}\right)}_{s}{\left(A^{-}\right)}_{n}{\left(\mathrm{Solvent}\right)}_{m}\right]}_{\mathrm{gel}}+n{\left(e^{-}\right)}_{\mathrm{metal}}\\ \mathrm{Neutral}\;\text{chains} & & \text{Oxidized}\;\text{chains} \end{array}$$
- **prevailing cation interchange**: The material shrinks during oxidation and swells during reduction (families (c) and (e)) [55–58]:
  (2) $$\begin{array}{ccc} {\left[({pPy}^{0}){\left({MA}^{-}\right)}_{n}{\left(C^{+}\right)}_{n}\right]}_{s} & \overset{\text{Re}d/\mathrm{Oxid}}{↔} & {\left[({pPy}^{n+}){\left({MA}^{-}\right)}_{n}\right]}_{s}+n{\left(C^{+}\right)}_{\mathrm{sol}}+n{\left(e^{-}\right)}_{\mathrm{metal}}\\ \mathrm{Neutral}\;\text{chains} & & \text{Oxidized}\;\text{chains} \end{array}$$

Where *s* means solid and *sol* is solution, *MA*^−^ represents any charge balancing macro-anions trapped inside the CP during polymerization, *pPy* represent the polypyrrole (or any other CP) chains and C^+^ represent a cation. The role of the solvent molecules in this case is not clear, due to the permanent presence of ionic species in the material interacting with the solvent dipolar moment. Reactions (1) and (2) produce positive charges on the polymeric chains: those processes are also named ‘p- doping’.

Some conducting polymers, as most of the polythiophene families, can also be reduced and reoxidized from the neutral state, storing negative charges (‘n-doping’) along the chains at high cathodic potential (stable electrolytes are required) interchanging balancing cations [59,60]:

(3) $$\begin{array}{lll} {\left({pTH}^{0}\right)}_{s}+n{\left(C^{+}\right)}_{\mathrm{aq}}+n{\left(e^{-}\right)}_{\mathrm{metal}} & ↔ & {\left[{\left({pTH}^{n-}\right)}_{s}{\left(C^{+}\right)}_{n}{\left(\mathrm{solvent}\right)}_{m}\right]}_{\mathrm{gel}}\\ \mathrm{Neutral}\;\text{chains} & & \;\;\text{Reduced}\;\text{chains} \end{array}$$

In general, two basic redox processes can occur from CPs:

(4) $$\begin{array}{ccccc} \text{Reduced}\;\text{chains} & ↔ & \text{Neutral}\;\text{chains} & ↔ & \text{Oxidized}\;\text{chains}\\ n\;\text{doping} & \left(a\right) & & \left(b\right) & p\;\text{doping} \end{array}$$

All of the above-described reactions are simplified versions. Any film of a conducting polymer establishes a physical equilibrium between solvent, anions and cations in the polymer matrix and in the electrolyte. So the charge balance is determined by simultaneous interchange of anions, cations and solvent across the polymer/solvent interface [61–64]. For each family one of the reactions (1) or (2) prevails. The final result of reactions (1), (2), or (3) is a polymeric salt, a polyelectrolyte.

## 4. Electrochemical Properties

Any of those electrochemical reactions promotes a continuous movement of anions or cations across the film/solution interface. The obtained polymeric salts are non-stoichiometric compounds [65,66]. The three considered reactions are reversible, so the stoichiometry of the material can be adjusted to any intermediate content between non-charged (reduced) polymer and fully oxidized polymer (around 50% of counterion content). Some properties change as a function of the material composition: conductivity, electro-chemo-mechanical effects (change of volume), electrochromic properties, charge storage, chemical storage and electroporosity [2–6,51,52]. These are described as follows:

- **Electrochemo-mechanical properties:** the entrance and expulsion of counterions and solvent from the solution, driven by the electrochemical reaction, promotes reversible changes on the material volume, (see Figure 4), which can be applied to generate macroscopic movement and mechanical energy [67–70].
- **Electrochromic properties:** the reversible reorganization of the double bonds along the polymeric chains generates and destroys chromophores (polarons and bipolarons) adsorbing light along the UV-vis and near IR regions of the spectra. The color of the material can be changed reversibly, by controlling the concentration of chromophores, under electrochemical reaction in a continuous and reversible manner [71–75].
- **Charge storage:** transition from neutral to oxidized polymers implies the storage of positive charges along the polymeric chains. Transition from neutral to reduced polymer implies the storage of negative charges along the polymeric chains. Therefore, CPs can be used as electrodic materials for polymeric batteries [76–78].
- **Porosity:** a film of a basic conducting polymer in a neutral state is a porous compacted structure, in which average distances between chains is short. During oxidation, coulombic repulsions among the emerging positive charges in neighboring chains increase the average distance between chains allowing counterions entrance [54,79–81].
- **Electron/Chemical transduction:** reverse electrochemical reactions are linked to the simultaneous interchange of chemical ions between the CP and the solution. This must be a univoque relationship; each injected electron forces the interchange of an one-valence chemical ion with the ambient electrolyte, suitable for a reversible storage and release of chemical and pharmacological compounds [82–84].

## 5. Multifunctional and Biomimicking Properties

The above described properties are linked to any of the electrochemical reactions: (1), (2) or (3), taking place in soft and wet materials, with a composition resembling that of animal organs: solvent, ions and polymeric (organic) molecules. Every property is linked to one or several similar functions occurring in biological organs [3,5,85–89], as shown in Table 1.

The electrochemical oxidation/reduction of any non-stoichiometric CP produces a multifunctional actuation of biomimetic properties. Only one reaction generates multiple properties.

## 6. Unparalleled Simultaneous Sensing Possibilities

The reverse electrochemical processes, (1), (2) or (3), suppose that any intermediate composition of the material is linked to an electrochemical equilibrium between: polymeric species, counterions and solvent inside the polymer; ions and solvent in solution, and electrons in the metal:

(5) $${\left[{\left({pPy}^{n+}\right)}_{s}{\left(A^{-}\right)}_{n}{\left(H_{2}O\right)}_{m}\right]}_{\mathrm{gel}}+n{\left(A^{-}\right)}_{\mathrm{sol}}+m(H_{2}O)↔{\left[{\left({pPy}^{(n+1)+}\right)}_{s}{\left(A^{-}\right)}_{n+1}{\left(\mathrm{solvent}\right)}_{m+a}\right]}_{\mathrm{gel}}+{\left(e^{-}\right)}_{\mathrm{metal}}$$

That means that any physical variable, such as mechanical (through ΔV), optical (through [pPy^n+^], polaronic and bipolaronic chromophores), electrical (applied i or E), magnetic (through [pPy^n+^], as radical-cations), *etc.*, or chemical variable, such as counterion concentration [A^−^]_aq_, ionic strength (I), or temperature (through the kinetic coefficient), acting on the electrochemical equilibrium, must promote a simultaneous energetic change of the electrons in the metal, which can be detected by means of a potentiostat. Working under equilibrium conditions the simplified expression of the Nerst equation as a function of the concentrations can be applied:

(6) $$E=E_{0}+\frac{R\cdot T}{n\cdot F}\;\text{ln}(\frac{{\left[{\left({pPy}^{(n+1)+}\right)}_{s}{\left(A^{-}\right)}_{n+1}{\left(\mathrm{solvent}\right)}_{m+a}\right]}_{\mathrm{gel}}}{{\left[{\left({pPy}^{n+}\right)}_{s}{\left(A^{-}\right)}_{n}{\left(\mathrm{solvent}\right)}_{m}\right]}_{\mathrm{gel}}\cdot {\left[A^{-}\right]}_{\mathrm{sol}}})$$

The sensing concept works under equilibrium, and under transition conditions. If we consider a single, lineal and ideal chain of conducting polymer (CP) constituted by *m* monomeric units, the reaction goes, through *n* consecutive oxidation equilibrium steps (*m* > *n*):

(7) $$\begin{array}{l} 1)\;\;\;\;\text{CP}+{\left(A^{-}\right)}_{\text{sol}}↔({\text{CP}}^{+})A^{-}+e^{-}\\ 2)\;({\text{CP}}^{+})A^{-}+{\left(A^{-}\right)}_{\text{sol}}↔({\text{CP}}^{2+})A_{2}^{-}+e^{-}\\ 3)\;({\text{CP}}^{2+})A_{2}^{-}+{\left(A^{-}\right)}_{\text{sol}}↔({\text{CP}}^{3+})A_{3}^{-}+e^{-}\\ 4)\;({\text{CP}}^{3+})A_{3}^{-}+{\left(A^{-}\right)}_{\text{sol}}↔({\text{CP}}^{4+})A_{4}^{-}+e^{-}\\ 5)\;({\text{CP}}^{4+})A_{4}^{-}+{\left(A^{-}\right)}_{\text{sol}}↔({\text{CP}}^{2+})A_{5}^{-}+e^{-}\\ \_\_\_\_\_\_\_\_\_\_\_\_\_\_\_\_\_\_\_\_\_\_\_\_\_\_\_\_\_\_\_\_\_\_\_\_\_\_\\ n)\;({\text{CP}}^{(n-1)+})A_{n-1}^{-}+{\left(A^{-}\right)}_{\text{sol}}↔({\text{CP}}^{n+})A_{n}^{-}+e^{-} \end{array}$$

The reaction goes on through *n* consecutive energetic conformational (electronic) states of the polymeric chain, with increasing conjugated planar segments. Each of those conformational electronic states establishes a fast electronic equilibrium, due to the high conductivity of the material, with electrons in the connecting metal. So, under transition conditions the electrode potential reflects the average conformational electronic state of the chains in the reacting film at any time during the reaction.

Both aspects, the slow chemical equilibrium and the fast electronic equilibrium, include the influence of all the above (reaction 5) chemical and physical variables on the electrode potential both, under equilibrium, or along the reaction. Consequently, any possible devices constructed on the bases of the described electrochemical properties are expected to work, simultaneously, as sensors of the ambient conditions [90–93]. Any of those devices should sense the ambient, while working, as the natural organs do, overcoming any unprecedented device that requires separate actuating and sensing systems with separate electrical connections.

The simultaneous actuating/sensing abilities of any electrochemical device based on conducting polymers (or similar organic materials as fullerenes, carbon nanotubes, graphenes,…) must be related to the sensing abilities of the self-supported material films under electrochemical reactions. This behavior will be illustrated for thick polypyrrole/DBSA films.

## 7. Film preparation of Polypyrrole in Dodecyl Benzene Sulfonate (DBSA) Aqueous Solutions

Polypyrrole films were prepared at room temperature (20 ± 2 °C) in dark conditions in a one-compartment electrochemical cell from an aqueous solution of 0.2 M DBSA and 0.2 M pyrrole. The working electrode was an AISI 316 stainless steel sheet, having a surface area of 5 cm^2^ on either side. Deposition was performed on both sides of the electrode. Two larger electrodes (10 cm^2^) of the same material were used as counter electrodes, placed symmetrically on both sides of the working electrode in order to obtain a uniform electrical field. A standard Ag/AgCl electrode from Metrohm was used as reference electrode.

**Figure 5.**
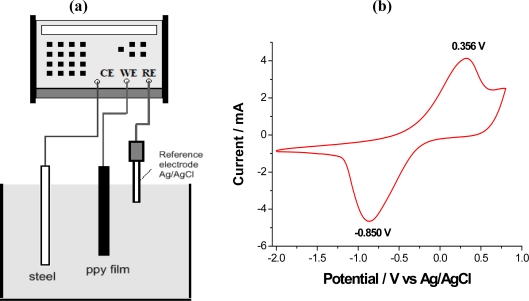
(a) Scheme of the electrochemical cell used to follow the electrochemical behavior of self-supported pPy/DBSA films. (b) Control voltammogram recorded at 6 mV s^−1^ between −2.0 and 0.80 V in 0.1 M LiClO_4_ aqueous solution at room temperature.

Polypyrrole (pPy) was electrogenerated by applying a constant anodic current density of 2 mA cm^−2^ during two hours. The overall charge consumed during the electropolymerization was 72.0 C. Two separate films were obtained, coating each of the electrode faces, with a mass of 32 ± 0.2 mg each, determined by means of a precision balance (±0.1 μg) by weight differences between coated and uncoated electrodes. After peeling from the working electrode, the films were submerged in de-ionized water for 24 hours to remove DBSA excess from the polymer surface. Film thicknesses of 85 ± 10 μm were measured using a COMECTA electronic digital micrometer with a precision of ±1 μm. All electrochemical studies were performed using an Autolab PGSTAT-100 potentiostat/galvanostat controlled by a personal computer using GPES electrochemical software. The electrochemical measurements were carried out in 0.1 M LiClO_4_ aqueous solutions, using the self-supported polymer film as the working electrode, a stainless steel counterelectrode and an Ag/AgCl reference electrode (Figure 5a). A Julabo T25 Cryostat/Thermostat (±0.1 °C) was used to study the influence of the temperature. All the other experiments were performed at 20 °C (room temperature).

### 7.1. Voltammetric Response

Figure 5b shows the experimental voltammogram recorded between +0.8 V, as anodic potential limit and −2.0 V, as cathodic potential limit, with 6 mV·s^−1^ the scan rate. Only one cathodic maximum, at −0.850 V, and one anodic maximum, at 0.356 V, are observed. The shape of the obtained voltammogram and peak potentials are similar to those reported for thinner pPy/DBS films [94–96] in aqueous solutions and quite different to those reported for pPy/ClO_4_^−^ films [97]. Here a larger potential separation between anodic and cathodic peaks is observed, which can be attributed to use of a thicker film. These results indicate that dodecyl benzene sulfonate (DBS^−^) is the prevailing counterion inside the material. Due to its large size, the counterion remains trapped, promoting an exchange of Li^+^ cations predominately during oxidation/reduction processes. This interchange will promote the insertion of cations and polymeric swelling during reduction and the cations expulsion with polymeric shrinking during oxidation. These volume changes were confirmed by the sense of the angular movements of a pPy/tape actuator under the flow of anodic and cathodic currents.

The electrochemical reaction can be written in a simplified form as:

(8) $${\left(\text{pPy}\;\text{chain}\right)}_{s}{\left({\text{DBS}}^{-}{\text{Li}}^{+}\right)}_{n}↔(\text{pPy}\;{\text{chain}}^{n+}){\left({\mathrm{DBS}}^{-}\right)}_{n}+n\;{\text{Li}}^{+}n\;e^{-}$$

The reaction occurring from left to right is the anodic process in which electrons are extracted from the polymeric chains. In Figure 5b, this process occurs during the anodic currents giving the maximum at 0.356 V. The charge extracted during oxidation can be obtained by integration of the maximum. The reaction from the right side towards the left side is the cathodic process, that is, electrons are introduced into the polymeric chains eliminating positive charges. This process is related to the cathodic currents originating the maximum at −0.850 V (negative currents on the voltammogram). The charge injected during reduction can be obtained by integration of this maximum.

### 7.2. Sensing Abilities under Constant Current

**Figure 6.**
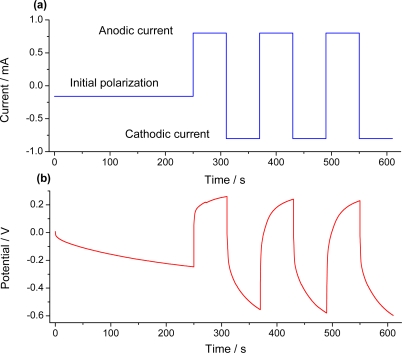
(a) Scheme of the applied current and (b) potential responses to the applied currents during film oxidation (shift to positive potentials) or reduction (shift to negative potentials).

In order to study the response of the material to different experimental variables, a galvanostatic procedure (Figure 6) was designed.

After stabilization of the initial oxidation state by applying a constant current of −0.150 mA for 250 s, the material film was submitted to three consecutive square waves of current (Figure 6a). The chronopotentiometric responses obtained during consecutive oxidation/reduction processes were recorded (Figure 6b).

**Figure 7.**
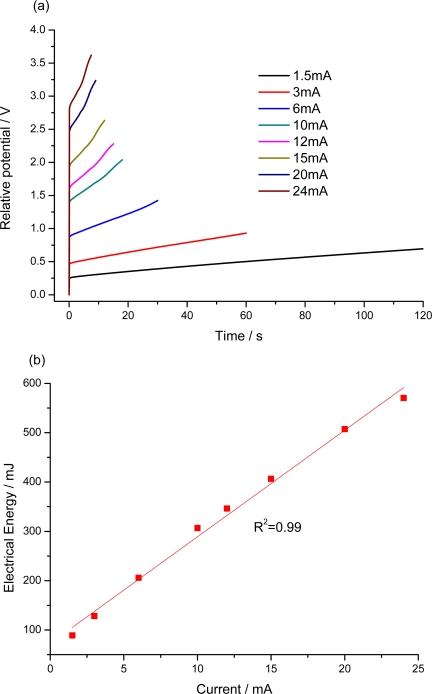
(a) Chronopotentiograms obtained for different anodic current pulses applied to a self supported pPy/DBSA film, flowing a constant charge of ±180 mC, in 0.1 M LiClO_4_ aqueous solution and (b) variation of the consumed electrical energy as a function of the applied current. R^2^ is the correlation coefficient of the linear fit.

The pPy/DBS film was submitted to different square waves of current ranging from ±1.5 to ±24 mA, passing a constant charge of ±180 mC. Figure 7 shows the obtained anodic chronopotentiograms. The potential evolves at higher potentials for higher currents. The higher initial step of the potential for rising currents is related to the different resistances present in the system: film resistance, interface resistance due to ions interchange, solution resistance and reaction resistance. After this initial change, the potential increases for rising anodic currents following the electrode processes. The electrical energy (*E*_e_) consumed by the polymer film during each oxidation/reduction was calculated as *E_e_* = *i*·∫*E*·*dt*, with *i* the constant current, *E* the electrodic potential at any time, *t*, of the current flow. Figure 7b shows the variation of the electrical energy as a function of the applied current. A linear fit was obtained for both cathodic and anodic processes: the material under reaction senses the applied current.

### 7.3. Temperature Sensor

**Figure 8.**
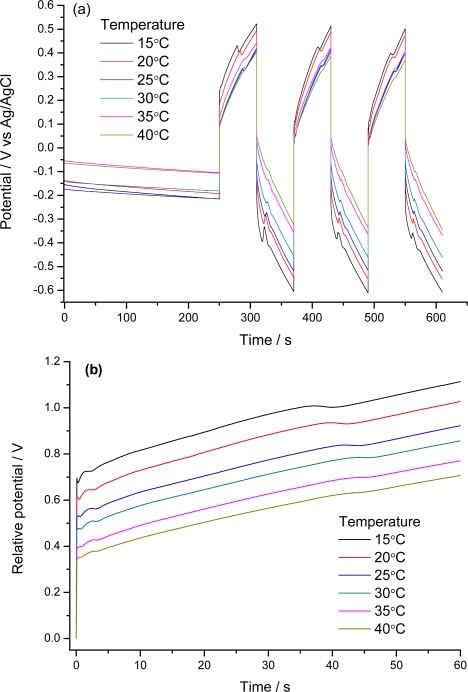
(a) Chronopotentiograms obtained following the scheme form Figure 6a at different temperatures by flow of ±4 mA during 60 s per step through a self-supported film of pPy/DBSA in 0.1 M LiClO_4_ aqueous solution. (b) The third oxidation chronopotentiograms.

**Figure 9.**
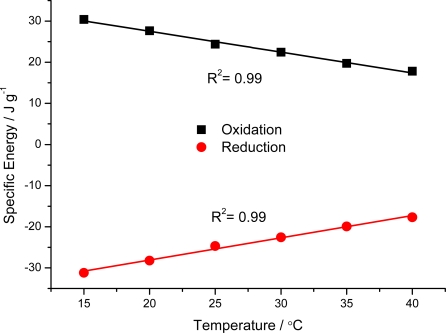
Variation of the electrical energy consumed during oxidation or reduction of a pPy/DBSA film during 60 s in 0.1 M LiClO_4_ aqueous solution as a function of the temperature. R^2^ is the correlation coefficient of the linear fit.

In reaction (1) or (2), as for any other chemical or electrochemical process, the reaction rate (anodic or cathodic) is expected to increase for rising experimental temperatures, due to the Arrhenius dependence of the reaction kinetic coefficient with T. This is equivalent to saying that working at a constant reaction rate, under flow of a constant current; the reaction is expected to occur with a lower reaction resistance (that means lower potentials) for increasing temperatures. In order to gain more knowledge into the sensing abilities of the electroactive material, the film is submitted to square current waves (±4 mA for 60 s every current) at different temperatures (15, 20, 25, 30, 35 and 40 °C). Figure 8a shows the obtained chronopotentiograms in which the evolutions of the reactive film potential for the different temperatures were overlapped. Figure 8b shows the response for the last oxidation step referred to the same arbitrary zero initial potential, in order to obtain the consumed electrical energies by integration of the curves. As expected, for the anodic processes decreasing potentials are observed for the same times of current flow and increasing temperatures. Rising available thermal energies require lower consumption of electrical energies during the reaction. Under constant current this produces lower potentials. Figure 9 shows the linear increase of the consumed electrical energy for decreasing working temperatures [92]. As a partial conclusion the electroactivity of the material, understood as electrochemical reactions of polymeric oxidation or reduction, act as a temperature sensor.

### 7.4. Concentration Sensor

**Figure 10.**
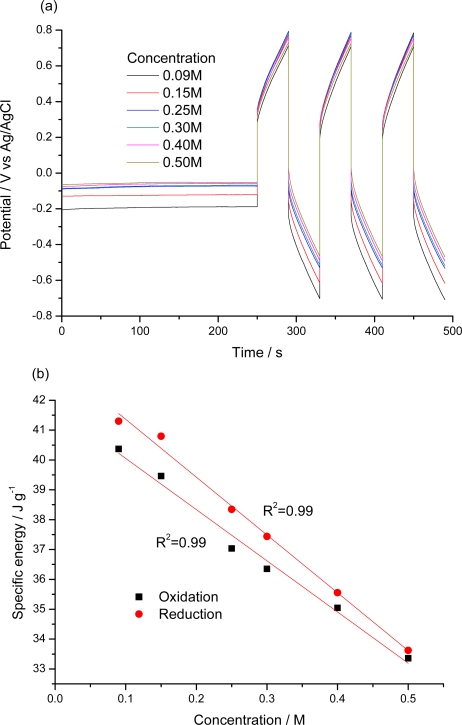
(a) Chronopotentiograms obtained from pPy/DBSA self-supported films for different LiClO_4_ concentrations. After stabilization of the initial oxidation state by applying a constant current of −0.01 mA for 250 s, square current waves of ±2.25 mA flowing for 40 s per step, are applied to a pPy/DBSA film. (b) Variation of the electrical energy as a function of the electrolyte concentration. R^2^ is the correlation coefficient of the linear fit.

Similar experiments were performed by changing the electrolyte concentration (0.01, 0.02, 0.05, 0.1, 0.25, 0.5 and 1 M) as shown in Figure 10. For the same time of a constant current flow, decreasing electrode potentials are observed for rising electrolyte concentrations. The effect increases when referred to the same arbitrary zero initial potential. The consumed specific electrical energy changes as a function of the concentration. (Figure 10b) We conclude that the material works as a sensor of the salt concentration in the ambient.

As final conclusions, we can state that the specific energy consumed during oxidation or reduction reactions of the studied film sense the influence of the variables: flowing current, working temperature or electrolyte concentration, on the system. The consumed electrical energy is a linear function of the studied variable.

### 7.5. Expected Sensing Electrochemical Devices

Those facts described in Sections 7.2 to 7.4 suggest that any device based on an electrochemical property (on the electrochemical reactions) of intrinsically conducting polymers is expected to act simultaneously as a sensor of any ambient and working variables acting on the reaction. Artificial muscles, polymeric batteries, smart membranes, drug delivery devices, electrochromic devices, among others, are expected to wear those simultaneous sensing-actuating properties. The two connecting wires should include both, actuating (*i.e.*, the current) and sensing (*i.e.*, the potential evolution) signals. We will focus our attention here on the present state of the art for those simultaneous actuating/sensing abilities of conducting polymers as reactive materials when used to construct artificial muscles.

## 8. Muscles and Artificial Muscles

Muscles are efficient devices working at constant temperature to transform chemical energy from glucose, into mechanical energy and heat. The fact that they work under constant temperature, far away from the servitude imposed by the Carnot’s principle, makes natural muscles much more efficient than any parallel human-made machine. Moreover, internal combustion devices, steam engines or turbo-reactors produce quite rudimentary movements, noise (acoustic and electromagnetic) and ambient deterioration, even though very useful for human development [98–100].

The actuation of any natural muscle is based on molecular motors constituted by polymeric chains of actin and myosin. The basic structure for the actuation is the sarcomere, where actin and myosin chains are organized as quasi-longitudinal fibers perpendicular to the sarcomere walls. The actuation mechanism of those electro-chemo-mechanical devices was described above.

### 8.1. Artificial Muscles

In order to mimic natural molecular motors, we shall attempt to include, at least, electric pulses and polymeric chains. Some pioneering devices were constructed in the 1950s using films of polymeric gels immersed in aqueous solutions [101–104]. At the beginning of the 1990s, a fast development of devices based on the interaction between electric fields, or electric currents, and polymers took place stimulated by the interest to reproduce commercial piezoelectric or electrostrictive (electromechanical) devices developed with inorganic materials, but using now similar properties from polymeric materials. The beginning of this explosive interest overlapped the discovery of intrinsically conductive polymers and the reverse electrochemical variation of their volume (Figure 4). This controlled volume variation envisaged the construction of new electrochemo-mechanical devices [67,105–107].

### 8.2. Classification of the Polymeric Artificial Muscles

Any developed device based on the interaction between electricity and polymers uses to be named artificial muscle. We can summarize the present state of the named artificial muscles, which actuation involves polymers, electric fields or electric currents, by their classification in two main areas:

- **Electromechanical actuators**: Artificial muscles responding mainly to electric fields, E (V), being the dimensions variation of the electroactive polymer proportional to:
  E^2^: Electrostrictive actuators
  E: Piezoelectric actuators
    Ferroelectric actuators
    Electrostatic actuators
    Electrokinetic actuators (electroosmotic)
- **Electrochemomechanical devices**: Artificial muscles responding mainly to electric charges, Q (mC), being the dimensions variation under control of the electrochemical reaction, and proportional to:
  Q: Electrochemical actuators

**Figure 11.**
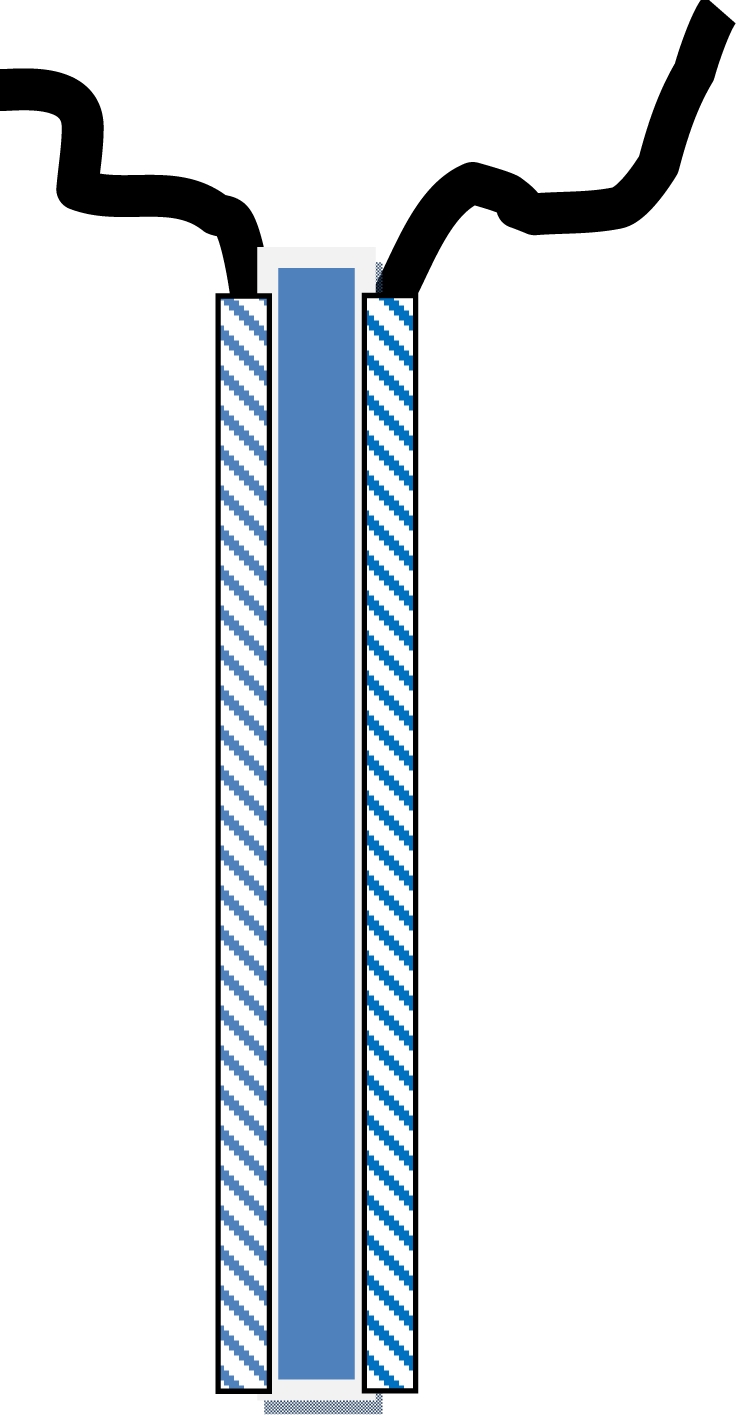
Three-layer structure for bending polymeric actuators. In electromechanical (EM) actuators, the electroactive polymer constitutes the internal layer, being the two external sputtered metals or electronic conductors. In electrochemomechanical (ECM) actuators, two films of reactive conducting polymers (electroactive polymers) constitute the external layers supported by an internal polymeric, adherent, flexible, non-conducting (or ionic conducting) film.

Conventionally electromechanical devices have been manufactured as bending thin films of dry polymer (named electroactive material), both sides coated with a very thin metallic film required to apply the electric field. Any actuator has a triple layer structure: metal/polymer/metal (Figure 11). Linear devices from materials having a large dimension’s change with the electric field are also constructed.

Bending electrochemomechanical actuators can be manufactured as bilayers, using a metallic electrode as counterelectode to allow the current flow, or as three-layers (see below), including working and counterelectrode. In the three-layers structure, the two external films are electroactive (react electrochemically, changing volume) during current flow. The internal film is an adherent, flexible and non-conducting (or ionic conductor) polymeric film.

In the case of linear devices, they can be made of fibers of conducting polymers [108–112], or by electropolymerization of a conducting polymer on springs of helical metallic wires until the generation of a tube, or on zigzag metal wires to generate films [110,113–117]. Bundles of films or fibers are checked to produce lineal displacements of weights [118]. Origami structures form films also provide good linear movements [119]. Different models have been proposed to describe mechanical behavior or electro-chemomechanical deformations [120–122] of this kind of devices.

### 8.3. Electrochemomechanical Muscles: Volume Variation

Artificial muscles have been developed from films of CP involving electrochemical reactions (1) or (2) as origin of the volume variations. When the interchange of anion prevails (accepted for families a, b, d, e and g) during the redox processes, the volume of the film swells during oxidation and shrinks during reduction. Under prevailing cation interchange (accepted for families c and e), the material shrinks during oxidation and swells during reduction. The important role played by the solvent interchange in both oxidized and reduced materials, is an unsolved point. Dimensional changes parallel to electrochemical reactions, have been followed by different methodologies, or estimated from experimental densities and weights of dried oxidized or reduced films from the pioneering works of the electrochemistry of conducting polymers [123,124]. Those changes were confirmed at microscopic level by “*in situ*” AFM [125–127], ellipsometry [128], conductivity [105] measurements, *in situ* electrogravimetry [129,130] and others [131,132].

### 8.4. Electrochemical Basic Molecular Motors and Muscle Similitude

**Figure 12.**
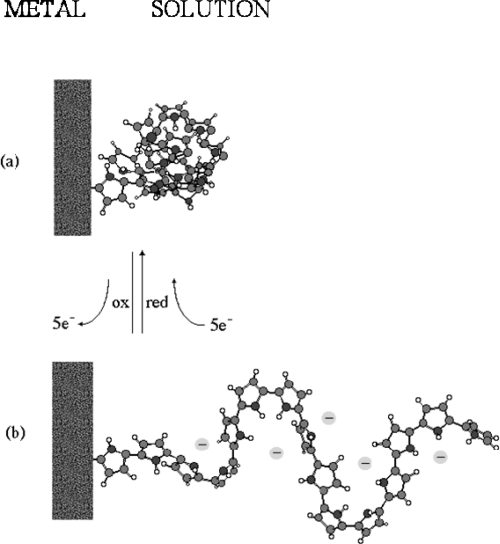
Molecular motor: reverse conformational changes (mechanical energy) stimulated by oxidation or reduction of the polymeric chain in an electrolyte. (a) Reduced chain, (b) Oxidized chain (adopted from Reference [3] with kind permission from Springer Science and Business Media Media).

We can imagine an ideal and lineal and neutral polymeric chain connected to a metallic electrode and immersed in an electrolyte (see Figure 12). The strong intramolecular interactions originate a coil compact structure of the chain (Figure 12a). Under oxidation changes on the double bonds distribution and storage of positive charges originate conformational movements until a stick-like structure (Figure 12b). This basic molecular actuator [133,134] working in a reversible way driven by electrochemical reactions includes: electric pulses, ions and solvent interchanges between the polymer and the solution, chemical reactions, stimulation of the conformational movements along polymeric chains and changes in the inter- and intramolecular interactions. Those processes occurring in soft and wet materials mimic, at molecular level, the consecutive events involved on the actuation of a natural anisotropic muscle. At the moment it is not known how to construct artificial sarcomere like structures (brushes) with chains of conducting polymers between two metallic (or electronically conducting) films. Only three-dimensional isotropic, microscopic and stable changes of volume are available with films of conducting polymers under electrochemical reactions.

### 8.5. Devices Giving Macroscopic Movements

**Figure 13.**
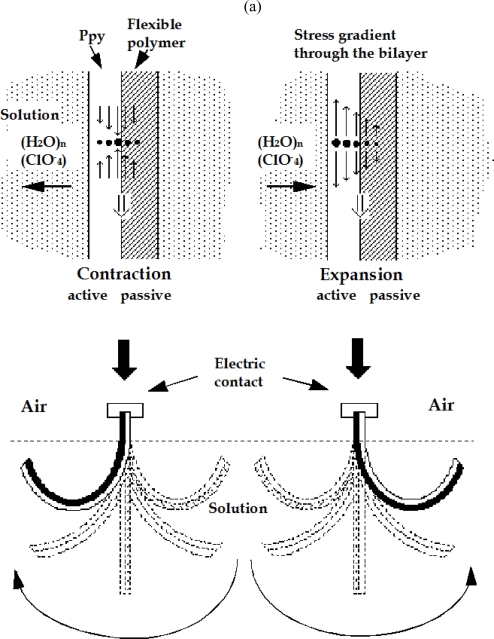

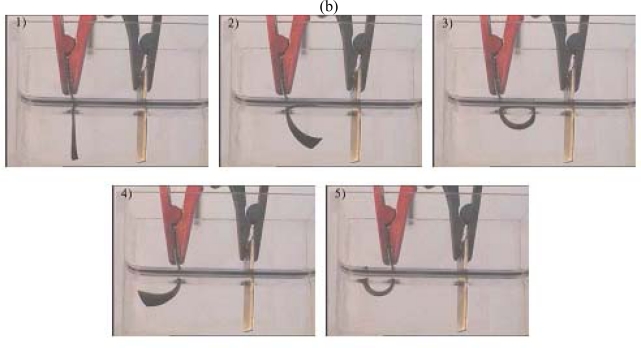
(a) Polypyrrole/tape bilayer. Induced stress gradients by electrochemical reactions (Reproduced with kind permission from Springer Science and Business Media [2]). (b) Angular movement described by the free end of a bilayer muscle (CP–tape) under a current flow of 15 mA (1, 2 and 3), or of 15 mA (4 and 5), the muscle being immersed in a 0.1 M aqueous electrolyte (adopted from References [2,52] with kind permissions from Springer Science and Business Media Media and Marcel Dekker Inc).

Bilayer structures of conducting polymer (CP) film/adherent polymer film [67,106,107,135,136], CP/metal [137–139], CP/solid state electrolyte [140], CP/CP [141,142], CP/plastic [143], CP/paper or CP/thin film of any flexible material metal coated (*i.e.*, by sputtering) [144] have been elegant solutions to translate the microscopic changes of volume taking place in films of CP into increasing anisotropic mechanical stress gradient across the bilayer interface, generating an uniform macroscopic bending movements (Figure 13a). The presence of a metallic counterelectrode is required to allow the current flow, originating the electrochemical reaction that produces the bending movement. However, an important fraction of the consumed electrical energy is wasted to produce the electrochemical reactions occurring at the counterelectrode (such as solvent dissociation, which requires a high overpotential). Moreover those reactions will generate new chemicals and pH variations, migrating until the muscle and promoting the progressive deterioration of the actuating film.

The construction of a triple layer CP/tape/CP [107] allows a more efficient actuation by using the same current two times to produce opposite volume changes and avoiding the use of metallic counterelectrodes (Figure 11). One of the CP films, the working electrode (WE) acts as the anode, swells (for preferential anionic interchange during the reaction) and pushes the device. The second CP film is the counterelectrode (CE) acts as the cathode shrinks and trails the device. The CE uses to be short-circuited with the reference electrode (RE) output of the potentiostat. On this way we follow the instantaneous potential difference between the two CP films: the muscle potential. The muscle potential evolution under flow of a constant current is a result of the two electrochemical reactions: oxidation at the anode and reduction at the cathode. Consequently we expect those actuating reactions to be influenced by the ambient variables, becoming a sensor of those variables.

The three layers muscles can work outside the liquid electrolytic media using an ionic conducting membrane separating the two films of CP. This membrane can be obtained by solvent evaporation and UV irradiation [72,145,146], or by formation of interpenetrated networks [147–152]: the two films of CP are generated by chemical polymerization on the external part of the membrane film.

Different structures can be produced by combination of several double or triple layers to transform bending into linear movements, each of those structures including WE, CE and reference electrode [153–155].

The actuating films of CP can be generated by electropolymerization. Electrogeneration is compatible with micro and nanotechnologies [156] giving elegant and imaginative microdevices[157] and microtools constituted by bending bilayers [69,137–139,158–165] or trilayers [166].

### 8.6. Electrochemical Nature of the Movement

**Figure 14.**
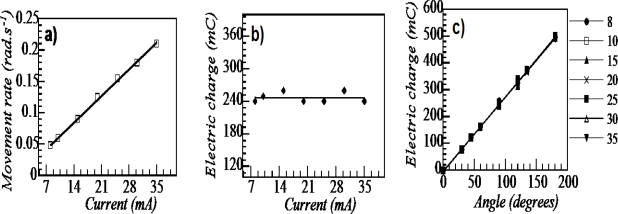
(a) Linear relationship between the applied current and the angular rate determined from the times required to describe an angular movement of 90 degrees under seven different currents (b) Electrical charge consumed by the triple layer muscle to move through 90 degrees. (c) Electrical charge consumed by the triple layer muscle to describe different angles (30, 45, 60, 90, 120, 135, and 180 degrees) under the different currents studied. Experiments in 1 M LiClO_4_ aqueous solution (adopted from Reference [167] with kind permission from The Royal Society of Chemistry).

Volume changes per unit of time, and the produced macroscopic movement, are expected (Equation 1) to be under control of the driving current. The Faraday’s expression should quantify the amount of interchanged ions. For driving current ranging from 8–35 mA, Figure 14a shows the experimental linear relationship between the driving current and the produced angular movement rate:

(9) $$\begin{array}{c} \text{Angular}\;\text{movement}\;\text{rate}\;(\text{rad}\;s^{-1})=\text{slope}\;(\text{rad}\;s^{-1}\;{\text{mA}}^{-1})\;x\;\text{applied}\;\text{current}\;(\text{mA})\\ \omega =k\cdot i \end{array}$$

Whatever the applied current, the experimental slope is a constant, as expected for an electrochemically driven motor. Moreover, the experimental charge consumed to perform an angular movement of one degree is constant (Figure 14c) due to the overlapping evolution of the described angle as a function of the consumed charge for different driven constant currents:

(10) $$\begin{array}{c} \text{Described}\;\text{angle}\;(\text{degrees})=\text{slope}\;(\text{degree}\;{\text{mC}}^{-1})\;x\;\text{consumed}\;\text{charge}\;(\text{mC})\\ \alpha =\omega \cdot t=k\cdot i\cdot t=k\cdot q \end{array}$$

Consequently, whatever the testing current the same charge is consumed in order to perform the same angular movement (Figure 14b). Equation 10 is a translation of the Faradays laws to bending electro-chemo-mechanical artificial muscles. The charge quantifies the amount of counterions interchanged, so the composition variation, the volume variation of the CP film and the stress gradient at the interface between the polymer films.

The expressions (9) and (10) can be applied to bilayers, triple-layers or complex structures [91,92,167–169]. Figure 14 quantifies the electrochemical nature of the movement. For any specific muscle, we can define the charge required to describe an angular movement of one degree (**a** mC degree^−1^). From **a**, the charge Q required to attain any new position (any new angle) from the actual position can be obtained:

(11) $$Q(\text{mC})=a(\text{mC}\;{\text{degree}}^{-1})\cdot \text{angle}\;(\text{degree})=a\cdot \alpha$$

This is an electrochemo-positioning device. Once defined the charge, Q, required to describe a defined angle, e.g., 90 degrees, the time consumed for the movement is under control of the charge flowing through the device per unit of time: the current [2,168].

**Figure 15.**
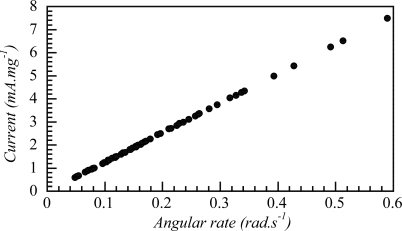
Angular rate measured through a movement of 90 degrees using a triple-layer muscle of different dimensions (including different weights of polypyrrole films: 8.3, 7.8, 7.4, 6, 5.5, 5.1, 5, 4, 3.7, 3.5, 3, 2.3 and 2 mg) in 1M LiClO4 aqueous solution under different currents (10, 15, 20, 25 and 30 mA) (adopted from Reference [167] with kind permission from The Royal Society of Chemistry).

The electrochemical nature of the device allows to state that different artificial muscles, having different surface area or constructed with pPy films having different film thickness (*i.e.*, weight), must produce the same angular movement rate under variation of the same composition gradient, by flow of analogous charge per unit of time (current) and per unit of CP weight (same variation of the oxidation deep) as confirmed by Figure 15.

$$\omega /w\;(\text{rad}\cdot s^{-1}\cdot {\text{mg}}^{-1})=k\cdot i/w\;(\text{rad}\cdot s^{-1}{\text{mA}}^{-1})\;(\text{mA})\;({\text{mg}}^{-1})$$

Allowing a more general expression than equation 9:

(12) $$\Omega =\text{slope}\cdot i\cdot w^{-1}=\text{slope}\cdot q\cdot t^{-1}\cdot w^{-1}=\text{slope}\cdot j$$

where Ω is the angular rate (rad·s^−1^), i is the constant current (mA) flowing though the muscle and w is the weight of the conducting polymer films. Considering j as mC·s^−1^·mg^−1^ this expression states that the angular movement, Ω, of any artificial muscle constituted by conducting polymers is under control of the amount of charge flowing through the muscle per second and per weigh unit of the material. This j quantifies the variation of the oxidation rate (counterion’s composition change) per weight unit. As conclusion the angular movement for those artificial muscles is under control of the oxidation rate per unit of weight, as corresponds to any electrochemical device.

Thus, the electrochemical nature of the movement allows an excellent control of both the movement rate (by the applied current) and the angular position (by the charge). This means that we have a perfect electrical machine able to transform the electrical energy into mechanical energy. The electrochemically stimulated conformational movements of the polymeric chains and the concomitant changes of volume are used as transducers from the electrical energy to the mechanical energy.

### 8.7. Sensing Muscles

**Figure 16.**
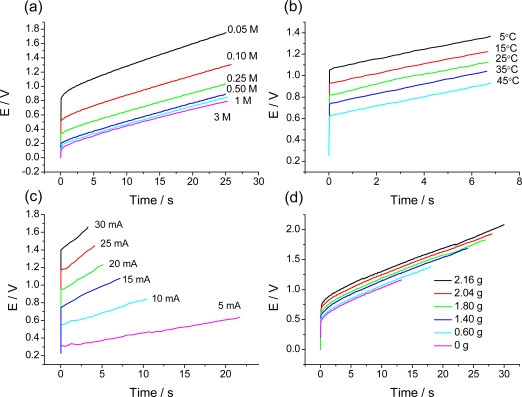
Chronopotentiograms obtained when a triple layer (2 × 1.5 cm^2^, 12 mg of PPy) describes 90° (a) in aqueous solutions of LiClO_4_ (3, 1, 0.5, 0.25, 0.1, and 0.05 M) under a constant current of 10 mA, (b) in 0.1 M LiClO_4_ at different temperatures: 5, 15, 25, 35 and 45 °C. (c) Under flow of different currents: 5, 10, 15, 20, 25, and 30 mA. (d) Shifting different attached steel weights: 0.6, 1.4, 1.8, 2.04, and 2.16 g with a device of 12 mg of polymer weight (adopted from References [170,171]).

**Figure 17.**
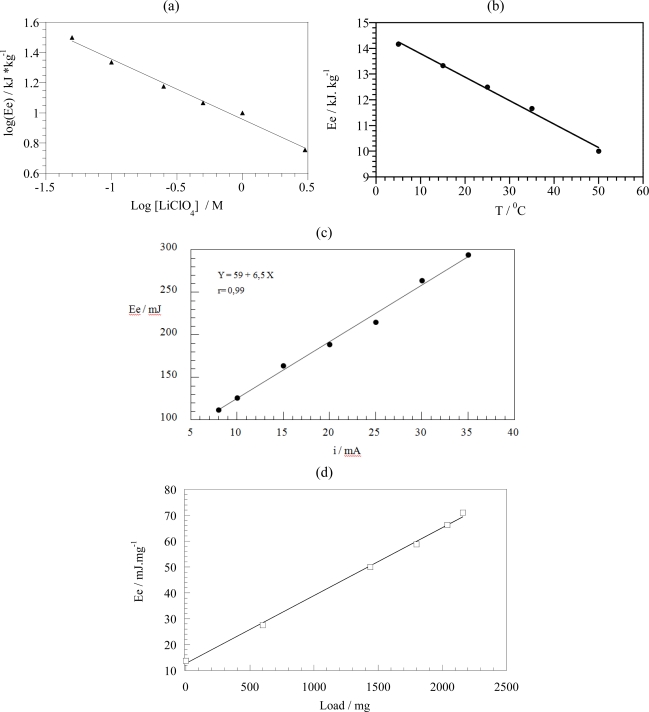
Consumed electrical energy in Figure 16 by the device as a function of the different studied variables: (a) electrolyte concentration, (b) temperature, (c) current, and (d) shifted weight (adopted from References [170,171]).

Based on the electrochemical nature of the movement, it should be expected that any mechanical, electrical, optical, thermal, magnetic or chemical variable acting on the reaction must influence the potential evolution of the working device. This should be a specific application to artificial muscles of the above described sensing abilities of the reactive material. Rising values of those variables increasing the reaction rate (electrolyte concentration or temperature) will promote an evolution of the muscle potential while moving along the same angle at lower values of the potential for the same time of current flow (Figure 16). Those variables which increase will decrease the reaction rate (mechanical stress) will promote a shift of the potential evolution while moving at higher muscle potentials [90–92,155,167–171]. Both, the muscle potential at a defined time of current flow, and the consumed electrical energy (i∫E dt) change linearly as a function of the different variables. (see Figure 17). Figures 16d and 17d indicate how the working muscle sense the different weights of steel plates adhered to the bottom of the muscle and trailed during the angular movement.

### 8.8. Tactile Muscles

**Figure 18.**
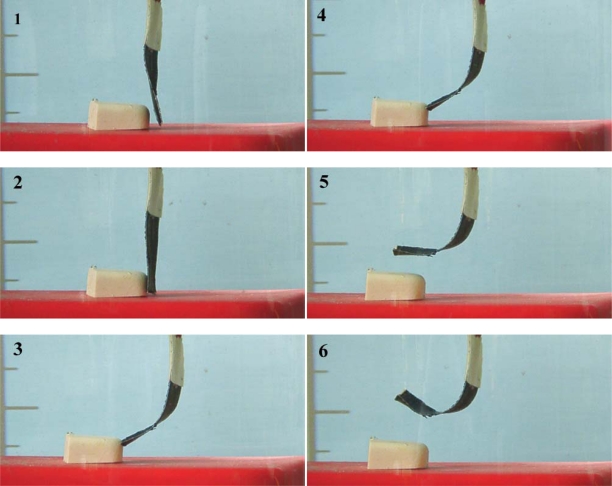
(1) The triple layer muscle initiates its movement under a constant current of 5 mA, in 1 M LiClO_4_ aqueous solution; (2) 10 s later; (3), (4) the muscle meets the obstacle weighing 6,000 mg, pushing and sliding it; (5) the angular movement allows the muscle to overcome the border of the obstacle; (6) the free movement continues until the current stops. The original position (1) is recovered by flow of -5mA during the same time (adopted from Reference [173] with kind permission from Wiley Interscience).

**Figure 19.**
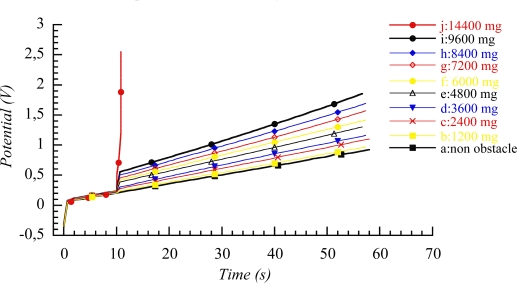
Chronopotentiograms obtained from a triple layer macroscopic muscle containing two polypyrrole films [2 cm × 1.5 cm × 13 μm] weighing 6 mg each, under flow of 5 mA in 1 M LiClO_4_. The muscle moves freely, contacting an obstacle after 10 s and sliding it for 3.5 s, overcomes its border and continues with a full angular movement of 108° (from −18° to +90°). The initial position is recovered by applying a current of −5 mA for 57 s. Obstacles weighing 1.2, 2.4, 3.6, 4.8, 6.0, 7.2, 8.4, 9.6 mg were slid, but the muscle was unable to push and slide an obstacle weighing 14.4 g (adopted from Reference [173] with kind permission from Wiley Interscience).

The mechanical sensing characteristics (increase of the muscle potential when the trailed weight rises) announce the possibility to develop a tactile sensor. If an obstacle is located in the muscle’s pathway (Figure 18), before reaching the obstacle the muscle moves freely, under constant current: the evolution of the muscle potential overlaps that of the free muscle. When the muscle touches the obstacle, the mechanical resistance influences the anodic and cathodic electrochemical reactions occurring in the two constituent, anode and cathode, polymeric films. Consequently a step is observed on the muscle potential at the contact time, the potential step is proportional to the opposed mechanical resistance. Rising weights of the obstacle produce increasing potential steps (Figure 19). Both, potential step and consumed electrical energy after contact and during shifting times, follow a linear evolution as a function of the obstacle weight [172–174]. When the muscle is not able to shift the obstacle the potential steps by several volts. During actuation the muscle potential of the free muscle evolves from a few mV to hundred of mV. The muscle potential gradient at the contact time with the obstacle ranges from a few mV to several V. Actuating and sensing signals are of the same order of magnitude.

These results announce the development of a class of tools and robots where the electrical machines (actuators) are at the same time, and through the same two connecting wires, sensors of the working conditions and of the ambient variables. In contrast to conventional technologies, where actuators and sensors are independent tools, requiring different electrical connections, complexes interfaces with computers and complexes software to develop intelligent machines.

## 9. Limitations and Challenges

The field of electrochemomechanical actuators is an emerging field for reactive, soft, wet, non-stoichiometric (giant), multifunctional and biomimicking materials. Electrochemical properties and devices show unprecedented simultaneous actuating and sensing abilities. At short term different sensing devices can be constructed, such as: full polymeric batteries sensing the state of charge, the working temperature and the number of remaining living cycles and including ambient friend salts; smart electrochromic devices (windows, goggles, mirrors, *etc.*) sensing the transmission state, the working temperature, the remaining lifetime and the supported mechanical stresses; and smart membranes, or artificial glands for medical or agricultural dosage, or nervous interfaces able to sense different actuating and ambient conditions of work.

There exists some limiting conditions for a fast development of those new devices. The required theoretical models able to integrate Electrochemistry, Polymer Science, Mechanics and Thermodynamics, describing experimental results and predicting new results are starting to emerge. There exists a limited control of the polymerization processes (chemical or electrochemical) to produce tailor-made materials: the polymerization process coexists with degradation and cross-linking processes. The relative weight of the parallel reactions still has not been quantified.

Moreover, different experimental difficulties must be overcome. A triple layer muscle is at the same time a battery, but the electrical energy stored in a triple-layer muscle under actuation still cannot be recovered during the opposite movement.

But difficulties also mean opportunities. A new world of soft and wet, sensing and tactile (conscious) actuating biomimicking machines is a challenge for the intellectual energy and engineering ability of young scientists in the new emerging technological world.

## Figures and Tables

**Table 1.** Electrochemical properties of conducting polymers, related functions and minded biological organs.

**Property**	**Action**	**Inspired organ**
Electrochemomechanical	Change of volume	Muscles
Electrochromic	Change of color	Mimetic skins
Charge storage	Current generation	Electric organs
Electroporosity	Transversal ionic flow	Membrane
Chemical or pharmacological storage	Chemical modulation or chemical dosage	Glands
Electron/ion transduction	ΔV (Chem/Phys. properties)	Bio-sensors
Electron/neurotransmitter	Channel V action	Nervous interface
